# Pyridine-4-carbaldehyde–fumaric acid (2/1)

**DOI:** 10.1107/S1600536813013445

**Published:** 2013-05-22

**Authors:** Bhupinder Sandhu, Sergiu Draguta, Marina S. Fonari, Mikhail Antipin, Tatiana V. Timofeeva

**Affiliations:** a Department of Chemistry & Biology, New Mexico Highlands University, 803 University Avenue, Las Vegas, NM 87701, USA; b Institute of Applied Physics Academy of Sciences of Moldova, Academy str. 5, MD-2028 Chisinau, Republic of Moldova

## Abstract

In the title co-crystal, 2C_6_H_5_NO·C_4_H_4_O_4_, two crystallographically different hydrogen-bonded trimers are formed, one in which the components occupy general positions, and one generated by an inversion centre. This results in the uncommon situation of *Z* = 3 for a triclinic crystal. In the formula units, mol­ecules are linked by O—H⋯N hydrogen bonds.

## Related literature

For background to the synthetic procedure, see: Aakeroy *et al.* (2006 ▶); Desiraju (2003 ▶). For the use of pyridine-4-carboxaldehyde in cytokine suppressive drugs, see: Boehm *et al.* (1996 ▶). For adducts of neutral pyridine derivatives and neutral fumaric acid, see: Bowes *et al.* (2003 ▶); Aakeroy *et al.* (2002 ▶, 2006 ▶, 2007 ▶); Batchelor *et al.* (2000 ▶). For a related structure, see: Liu *et al.* (2003 ▶).
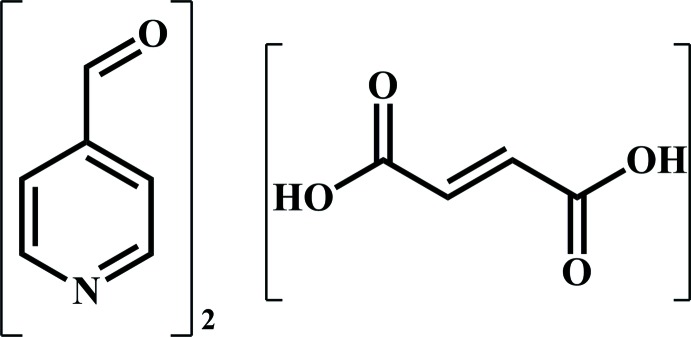


## Experimental

### 

#### Crystal data

2C_6_H_5_NO·C_4_H_4_O_4_
*M* *_r_* = 330.29
Triclinic, 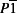

*a* = 6.9388 (12) Å
*b* = 10.1962 (18) Å
*c* = 17.002 (3) Å
α = 82.450 (3)°
β = 78.615 (3)°
γ = 80.064 (3)°
*V* = 1155.6 (4) Å^3^
*Z* = 3
Mo *K*α radiation
μ = 0.11 mm^−1^
*T* = 100 K
0.04 × 0.03 × 0.02 mm

#### Data collection

Bruker APEXII CCD diffractometer
Absorption correction: multi-scan (*SADABS*; Sheldrick, 2003 ▶) *T*_min_ = 0.996, *T*_max_ = 0.998
11918 measured reflections
5022 independent reflections
4351 reflections with *I* > 2σ(*I*)
*R*_int_ = 0.018

#### Refinement

*R*[*F* ^2^ > 2σ(*F* ^2^)] = 0.033
*wR*(*F* ^2^) = 0.094
*S* = 1.06
5022 reflections
337 parameters
H atoms treated by a mixture of independent and constrained refinement
Δρ_max_ = 0.30 e Å^−3^
Δρ_min_ = −0.24 e Å^−3^

### 

Data collection: *APEX2* (Bruker, 2005 ▶); cell refinement: *SAINT* (Bruker, 2001 ▶); data reduction: *SAINT*; program(s) used to solve structure: *SHELXTL* (Sheldrick, 2008 ▶); program(s) used to refine structure: *SHELXTL*; molecular graphics: *SHELXTL*; software used to prepare material for publication: *SHELXTL*.

## Supplementary Material

Crystal structure: contains datablock(s) global, I. DOI: 10.1107/S1600536813013445/gw2133sup1.cif

Structure factors: contains datablock(s) I. DOI: 10.1107/S1600536813013445/gw2133Isup2.hkl

Supplementary material file. DOI: 10.1107/S1600536813013445/gw2133Isup3.cml

Additional supplementary materials:  crystallographic information; 3D view; checkCIF report

## Figures and Tables

**Table 1:** Hydrogen-bond geometry (Å, °)

*D*—H⋯*A*	*D*—H	H⋯*A*	*D*⋯*A*	*D*—H⋯*A*
O9—H9*A*⋯N3	1.030 (19)	1.576 (19)	2.6047 (12)	176.1 (17)
O5—H5⋯N1	1.03 (2)	1.57 (2)	2.5952 (12)	172.0 (18)
O7—H7⋯N2	1.050 (19)	1.54 (2)	2.5826 (12)	172.9 (18)

**Fig. 1.**
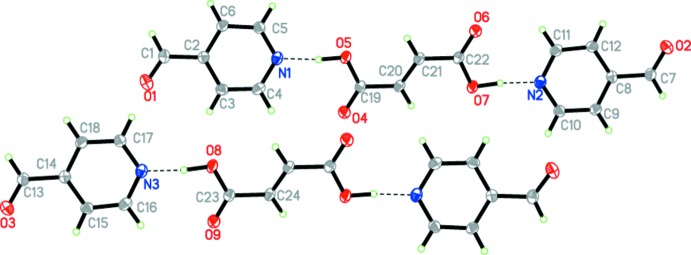
The molecular structure of (C6H5NO)2.(C4H4O4) showing (50%) probablity displacement ellipsoids and the atoms numbering scheme.

**Fig. 2.**
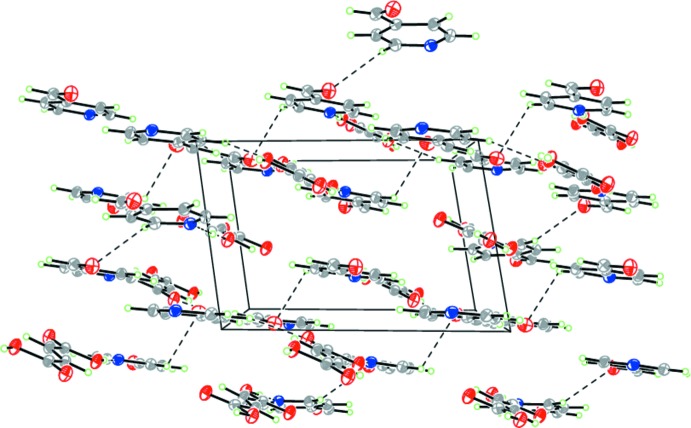
Crystal packing showing intermolecular hydrogen bonding interactions, view along c axis.

## supplementary crystallographic information

### Comment

The co-crystallization process is widely used to obtain new solid forms of active pharmaceutical ingredients (API) with enhanced physiochemical properties such as stability, dissolution rate, and solubility without altering their pharmacological behavior (Aakeroy *et al.* 2006; Desiraju, 2003). The pyridine-4-carboxaldehyde and fumaric acid are widely used in the biological and medicinal fields. Pyridine-4-carboxaldehyde is used as a starting material for the preparation of cytokine suppressive drugs to treat arthritis (Boehm *et al.* 1996). Fumaric acid is of interest since it is known to form supramolecular assemblies with N-aromatic bases (Batchelor *et al.*2000) and is generally regarded as safe (GRAS) in the list of pharmaceutically acceptable cocrystal formers. The asymmetric unit of the title compound contains three planar molecules of pyridine-4-carboxaldehyde, and one and a half molecules of fumaric acid. They comprise two crystallographically different H-bonded trimers (C_6_H_5_NO)_2._(C_4_H_4_O_4_), one of which occupies general position, while another resides on an inversion center in the triclinic unit cell as shown in Fig. 1. In the fumaric acid molecules, the C_22_—O_6_, C_19_—O_4_, and C_23_—O_8_ bond distances of 1.220 (3) Å, 1.219 (2) Å, 1.215 (3) Å are much shorter than the C_22_—O_7_, C_19_—O_5_, and C_23_—O_9_ bond distances of 1.312 (3) Å, 1.318 (2) Å, and 1.317 (2) Å respectively, indicating the neutral carboxyl groups in the crystal structure (Liu *et al.* 2003). However, the carboxylic O—H-atoms are on their way to the pyridine nitrogen atoms as it follows from the increased O—H distances in comparison with the standard values (0.86 Å).

The dihedral angles between the planar pyridine rings and the mean planes of fumaric acid molecules are 19.2° and 22.2° in the first, and of 25.7° in the second formula units in the crystal structure.

In the trimer, the neutral entities are held together *via* two (COOH) H···N (pyridine) hydrogen-bonds forming a complementary ADA array. The slightly corrugated aggregates are packed in stacks as shown in Fig. 2. The crystal packing is further stabilized by the weak C—H···O intermolecular interactions with participation of carbonyl oxygen atoms.

### Experimental

Pyridine-4-carboxaldehyde (19.3 µ*L*, 0.20 mmol) was dissolved in 5 ml of ethanol. To this solution was added fumaric acid (0.012 g, 0.10 mmol) in 5 mL of ethanol. The resulting solution was heated until the both compounds were dissolved completely and allowed to stand for slow evaporation. White prisms were obtained after 3 days. mp 215–220°C.

### Refinement

The hydrogen atoms of carboxylic groups of O5, O7 and O9 were localized in the difference-Fourier map and refined freely in isotropic approximation. The other hydrogen atoms were placed in calculated positions with C—H = 0.93 Å and refined in the riding model with fixed isotropic displacement parameters [*U*_iso_(H) = 1.2Ueq(C)].

### Crystal data

**Table:**

2C_6_H_5_NO·C_4_H_4_O_4_	*Z* = 3
*M**_r_* = 330.29	*F*(000) = 516
Triclinic, *P*1	*D*_x_ = 1.424 Mg m^−^^3^
Hall symbol: -P 1	Mo *K*α radiation, λ = 0.71073 Å
*a* = 6.9388 (12) Å	Cell parameters from 15620 reflections
*b* = 10.1962 (18) Å	θ = 2.5–30.8°
*c* = 17.002 (3) Å	µ = 0.11 mm^−^^1^
α = 82.450 (3)°	*T* = 100 K
β = 78.615 (3)°	Prism, white
γ = 80.064 (3)°	0.04 × 0.03 × 0.02 mm
*V* = 1155.6 (4) Å^3^	

### Data collection

**Table:**

Bruker APEXII CCD diffractometer	5022 independent reflections
Radiation source: fine-focus sealed tube	4351 reflections with *I* > 2σ(*I*)
Graphite monochromator	*R*_int_ = 0.018
φ and ω scans	θ_max_ = 27.0°, θ_min_ = 2.0°
Absorption correction: multi-scan (*SADABS*; Sheldrick, 2003)	*h* = −8→8
*T*_min_ = 0.996, *T*_max_ = 0.998	*k* = −13→13
11918 measured reflections	*l* = −21→21

### Refinement

**Table:**

Refinement on *F*^2^	Primary atom site location: structure-invariant direct methods
Least-squares matrix: full	Secondary atom site location: difference Fourier map
*R*[*F*^2^ > 2σ(*F*^2^)] = 0.033	Hydrogen site location: inferred from neighbouring sites
*wR*(*F*^2^) = 0.094	H atoms treated by a mixture of independent and constrained refinement
*S* = 1.06	*w* = 1/[σ^2^(*F*_o_^2^) + (0.0502*P*)^2^ + 0.2407*P*] where *P* = (*F*_o_^2^ + 2*F*_c_^2^)/3
5022 reflections	(Δ/σ)_max_ = 0.001
337 parameters	Δρ_max_ = 0.30 e Å^−^^3^
0 restraints	Δρ_min_ = −0.24 e Å^−^^3^

### Special details

**Table:**

Geometry. All e.s.d.'s (except the e.s.d. in the dihedral angle between two l.s. planes) are estimated using the full covariance matrix. The cell e.s.d.'s are taken into account individually in the estimation of e.s.d.'s in distances, angles and torsion angles; correlations between e.s.d.'s in cell parameters are only used when they are defined by crystal symmetry. An approximate (isotropic) treatment of cell e.s.d.'s is used for estimating e.s.d.'s involving l.s. planes.
Refinement. Refinement of *F*^2^ against ALL reflections. The weighted *R*-factor *wR* and goodness of fit *S* are based on *F*^2^, conventional *R*-factors *R* are based on *F*, with *F* set to zero for negative *F*^2^. The threshold expression of *F*^2^ > σ(*F*^2^) is used only for calculating *R*-factors(gt) *etc*. and is not relevant to the choice of reflections for refinement. *R*-factors based on *F*^2^ are statistically about twice as large as those based on *F*, and *R*- factors based on ALL data will be even larger.

### Fractional atomic coordinates and isotropic or equivalent isotropic displacement parameters (Å^2^)

**Table:**

	*x*	*y*	*z*	*U*_iso_*/*U*_eq_	
O1	0.03877 (12)	0.13984 (9)	0.93015 (5)	0.02957 (19)	
O3	0.32546 (12)	0.76849 (8)	0.60910 (4)	0.02667 (18)	
O2	0.31931 (13)	0.49716 (8)	−0.26052 (5)	0.02852 (19)	
O8	0.41401 (12)	0.85465 (8)	0.13133 (4)	0.02579 (18)	
O9	0.53088 (12)	1.02613 (8)	0.16686 (4)	0.02420 (18)	
H9A	0.497 (3)	0.9765 (19)	0.2235 (11)	0.069 (6)*	
O6	0.09363 (12)	0.27889 (8)	0.18081 (4)	0.02574 (18)	
O7	0.22987 (12)	0.45738 (8)	0.19320 (4)	0.02386 (18)	
O5	0.10344 (12)	0.24097 (8)	0.47468 (4)	0.02441 (18)	
H5	0.104 (3)	0.222 (2)	0.5358 (12)	0.076 (6)*	
O4	0.24013 (12)	0.41873 (8)	0.48845 (4)	0.02556 (18)	
N1	0.08206 (13)	0.17896 (9)	0.62885 (5)	0.02060 (19)	
N3	0.44019 (13)	0.91008 (9)	0.31230 (5)	0.02018 (19)	
N2	0.24664 (13)	0.50591 (9)	0.03924 (5)	0.02043 (19)	
C4	0.07788 (15)	0.27059 (11)	0.67926 (7)	0.0227 (2)	
H4	0.0919	0.3598	0.6573	0.027*	
C3	0.05391 (15)	0.24005 (11)	0.76207 (6)	0.0217 (2)	
H3	0.0500	0.3072	0.7964	0.026*	
C2	0.03570 (15)	0.10889 (10)	0.79403 (6)	0.0192 (2)	
C6	0.03953 (15)	0.01376 (10)	0.74200 (6)	0.0204 (2)	
H6	0.0265	−0.0763	0.7622	0.024*	
C5	0.06278 (15)	0.05330 (11)	0.65996 (6)	0.0208 (2)	
H5A	0.0652	−0.0115	0.6243	0.025*	
C1	0.01669 (15)	0.06833 (11)	0.88228 (6)	0.0231 (2)	
H1	−0.0145	−0.0185	0.9020	0.028*	
C16	0.40393 (15)	0.97924 (10)	0.37691 (6)	0.0214 (2)	
H16	0.4064	1.0730	0.3689	0.026*	
C15	0.36315 (15)	0.91885 (10)	0.45471 (6)	0.0207 (2)	
H15	0.3347	0.9706	0.4993	0.025*	
C14	0.36431 (14)	0.78107 (10)	0.46684 (6)	0.0184 (2)	
C18	0.40079 (15)	0.70913 (10)	0.40001 (6)	0.0206 (2)	
H18	0.4011	0.6151	0.4064	0.025*	
C17	0.43668 (15)	0.77782 (11)	0.32371 (6)	0.0216 (2)	
H17	0.4596	0.7293	0.2779	0.026*	
C13	0.33112 (15)	0.71201 (11)	0.55014 (6)	0.0211 (2)	
H13	0.3138	0.6205	0.5572	0.025*	
C11	0.23959 (15)	0.40809 (11)	−0.00530 (6)	0.0219 (2)	
H11	0.2156	0.3230	0.0213	0.026*	
C12	0.26613 (15)	0.42703 (10)	−0.08878 (6)	0.0206 (2)	
H12	0.2629	0.3558	−0.1191	0.025*	
C8	0.29763 (14)	0.55274 (10)	−0.12722 (6)	0.0188 (2)	
C9	0.30255 (15)	0.65446 (10)	−0.08099 (6)	0.0203 (2)	
H9	0.3224	0.7413	−0.1059	0.024*	
C10	0.27795 (15)	0.62679 (10)	0.00226 (6)	0.0205 (2)	
H10	0.2835	0.6957	0.0340	0.025*	
C7	0.32694 (15)	0.57964 (11)	−0.21668 (6)	0.0214 (2)	
H7A	0.3529	0.6659	−0.2405	0.026*	
C23	0.48373 (15)	0.95840 (10)	0.11436 (6)	0.0198 (2)	
C24	0.52458 (16)	1.02301 (11)	0.03024 (6)	0.0228 (2)	
H24	0.5885	1.1003	0.0201	0.027*	
C22	0.15105 (15)	0.34887 (10)	0.22167 (6)	0.0192 (2)	
C21	0.13369 (15)	0.31551 (10)	0.31054 (6)	0.0202 (2)	
H21	0.0709	0.2407	0.3347	0.024*	
C20	0.20063 (15)	0.38402 (10)	0.35802 (6)	0.0200 (2)	
H20	0.2633	0.4590	0.3340	0.024*	
C19	0.18294 (15)	0.35002 (10)	0.44683 (6)	0.0193 (2)	
H7	0.231 (3)	0.471 (2)	0.1308 (12)	0.077 (6)*	

### Atomic displacement parameters (Å^2^)

**Table:**

	*U*^11^	*U*^22^	*U*^33^	*U*^12^	*U*^13^	*U*^23^
O1	0.0306 (4)	0.0372 (5)	0.0216 (4)	−0.0025 (3)	−0.0053 (3)	−0.0080 (3)
O3	0.0327 (4)	0.0302 (4)	0.0173 (4)	−0.0076 (3)	−0.0034 (3)	−0.0010 (3)
O2	0.0369 (5)	0.0287 (4)	0.0214 (4)	−0.0058 (3)	−0.0054 (3)	−0.0065 (3)
O8	0.0331 (4)	0.0260 (4)	0.0196 (4)	−0.0131 (3)	−0.0037 (3)	0.0027 (3)
O9	0.0328 (4)	0.0262 (4)	0.0154 (4)	−0.0109 (3)	−0.0047 (3)	0.0004 (3)
O6	0.0345 (4)	0.0251 (4)	0.0205 (4)	−0.0114 (3)	−0.0067 (3)	−0.0005 (3)
O7	0.0316 (4)	0.0245 (4)	0.0168 (4)	−0.0131 (3)	−0.0033 (3)	0.0033 (3)
O5	0.0333 (4)	0.0244 (4)	0.0171 (4)	−0.0126 (3)	−0.0042 (3)	0.0029 (3)
O4	0.0322 (4)	0.0261 (4)	0.0211 (4)	−0.0108 (3)	−0.0063 (3)	−0.0016 (3)
N1	0.0189 (4)	0.0237 (5)	0.0186 (4)	−0.0052 (3)	−0.0027 (3)	0.0017 (3)
N3	0.0197 (4)	0.0228 (4)	0.0178 (4)	−0.0044 (3)	−0.0031 (3)	−0.0001 (3)
N2	0.0187 (4)	0.0232 (5)	0.0188 (4)	−0.0047 (3)	−0.0024 (3)	0.0009 (3)
C4	0.0221 (5)	0.0190 (5)	0.0273 (5)	−0.0056 (4)	−0.0058 (4)	0.0024 (4)
C3	0.0231 (5)	0.0199 (5)	0.0237 (5)	−0.0049 (4)	−0.0055 (4)	−0.0036 (4)
C2	0.0161 (5)	0.0222 (5)	0.0191 (5)	−0.0041 (4)	−0.0022 (4)	−0.0017 (4)
C6	0.0209 (5)	0.0185 (5)	0.0214 (5)	−0.0056 (4)	−0.0022 (4)	0.0002 (4)
C5	0.0216 (5)	0.0220 (5)	0.0191 (5)	−0.0052 (4)	−0.0019 (4)	−0.0035 (4)
C1	0.0215 (5)	0.0268 (5)	0.0196 (5)	−0.0030 (4)	−0.0021 (4)	−0.0006 (4)
C16	0.0233 (5)	0.0190 (5)	0.0222 (5)	−0.0054 (4)	−0.0039 (4)	−0.0011 (4)
C15	0.0217 (5)	0.0215 (5)	0.0198 (5)	−0.0052 (4)	−0.0031 (4)	−0.0041 (4)
C14	0.0161 (5)	0.0222 (5)	0.0175 (5)	−0.0056 (4)	−0.0029 (4)	−0.0006 (4)
C18	0.0218 (5)	0.0194 (5)	0.0214 (5)	−0.0047 (4)	−0.0040 (4)	−0.0030 (4)
C17	0.0219 (5)	0.0241 (5)	0.0197 (5)	−0.0041 (4)	−0.0033 (4)	−0.0050 (4)
C13	0.0205 (5)	0.0226 (5)	0.0202 (5)	−0.0060 (4)	−0.0033 (4)	0.0005 (4)
C11	0.0210 (5)	0.0201 (5)	0.0252 (5)	−0.0062 (4)	−0.0054 (4)	0.0023 (4)
C12	0.0214 (5)	0.0194 (5)	0.0227 (5)	−0.0052 (4)	−0.0058 (4)	−0.0022 (4)
C8	0.0161 (5)	0.0221 (5)	0.0182 (5)	−0.0032 (4)	−0.0032 (4)	−0.0017 (4)
C9	0.0216 (5)	0.0184 (5)	0.0208 (5)	−0.0048 (4)	−0.0035 (4)	−0.0002 (4)
C10	0.0213 (5)	0.0209 (5)	0.0192 (5)	−0.0038 (4)	−0.0027 (4)	−0.0028 (4)
C7	0.0226 (5)	0.0226 (5)	0.0188 (5)	−0.0040 (4)	−0.0038 (4)	−0.0008 (4)
C23	0.0186 (5)	0.0229 (5)	0.0174 (5)	−0.0036 (4)	−0.0029 (4)	−0.0002 (4)
C24	0.0281 (5)	0.0230 (5)	0.0185 (5)	−0.0109 (4)	−0.0035 (4)	0.0022 (4)
C22	0.0185 (5)	0.0200 (5)	0.0179 (5)	−0.0036 (4)	−0.0019 (4)	0.0008 (4)
C21	0.0211 (5)	0.0207 (5)	0.0180 (5)	−0.0060 (4)	−0.0019 (4)	0.0024 (4)
C20	0.0210 (5)	0.0194 (5)	0.0188 (5)	−0.0058 (4)	−0.0018 (4)	0.0022 (4)
C19	0.0183 (5)	0.0200 (5)	0.0188 (5)	−0.0039 (4)	−0.0022 (4)	0.0006 (4)

### Geometric parameters (Å, º)

**Table:**

O1—C1	1.2089 (14)	C16—C15	1.3810 (14)
O3—C13	1.2109 (13)	C16—H16	0.9500
O2—C7	1.2089 (13)	C15—C14	1.3917 (15)
O8—C23	1.2155 (13)	C15—H15	0.9500
O9—C23	1.3175 (13)	C14—C18	1.3909 (14)
O9—H9A	1.030 (19)	C14—C13	1.4898 (14)
O6—C22	1.2199 (13)	C18—C17	1.3890 (14)
O7—C22	1.3119 (12)	C18—H18	0.9500
O7—H7	1.050 (19)	C17—H17	0.9500
O5—C19	1.3171 (12)	C13—H13	0.9500
O5—H5	1.03 (2)	C11—C12	1.3866 (15)
O4—C19	1.2191 (13)	C11—H11	0.9500
N1—C5	1.3388 (14)	C12—C8	1.3921 (14)
N1—C4	1.3415 (14)	C12—H12	0.9500
N3—C17	1.3408 (14)	C8—C9	1.3897 (14)
N3—C16	1.3418 (13)	C8—C7	1.4892 (14)
N2—C10	1.3403 (14)	C9—C10	1.3888 (14)
N2—C11	1.3419 (14)	C9—H9	0.9500
C4—C3	1.3846 (15)	C10—H10	0.9500
C4—H4	0.9500	C7—H7A	0.9500
C3—C2	1.3928 (14)	C23—C24	1.4888 (14)
C3—H3	0.9500	C24—C24^i^	1.309 (2)
C2—C6	1.3896 (14)	C24—H24	0.9500
C2—C1	1.4890 (14)	C22—C21	1.4900 (14)
C6—C5	1.3856 (14)	C21—C20	1.3280 (15)
C6—H6	0.9500	C21—H21	0.9500
C5—H5A	0.9500	C20—C19	1.4897 (14)
C1—H1	0.9500	C20—H20	0.9500
			
C23—O9—H9A	108.3 (10)	O3—C13—C14	122.26 (10)
C22—O7—H7	107.3 (11)	O3—C13—H13	118.9
C19—O5—H5	109.4 (11)	C14—C13—H13	118.9
C5—N1—C4	118.60 (9)	N2—C11—C12	122.25 (9)
C17—N3—C16	118.84 (9)	N2—C11—H11	118.9
C10—N2—C11	119.23 (9)	C12—C11—H11	118.9
N1—C4—C3	122.46 (10)	C11—C12—C8	118.55 (9)
N1—C4—H4	118.8	C11—C12—H12	120.7
C3—C4—H4	118.8	C8—C12—H12	120.7
C4—C3—C2	118.67 (10)	C9—C8—C12	119.17 (9)
C4—C3—H3	120.7	C9—C8—C7	119.64 (9)
C2—C3—H3	120.7	C12—C8—C7	121.19 (9)
C6—C2—C3	119.03 (9)	C10—C9—C8	118.76 (9)
C6—C2—C1	119.58 (9)	C10—C9—H9	120.6
C3—C2—C1	121.37 (9)	C8—C9—H9	120.6
C5—C6—C2	118.45 (9)	N2—C10—C9	122.02 (9)
C5—C6—H6	120.8	N2—C10—H10	119.0
C2—C6—H6	120.8	C9—C10—H10	119.0
N1—C5—C6	122.79 (9)	O2—C7—C8	123.27 (10)
N1—C5—H5A	118.6	O2—C7—H7A	118.4
C6—C5—H5A	118.6	C8—C7—H7A	118.4
O1—C1—C2	123.58 (10)	O8—C23—O9	124.76 (9)
O1—C1—H1	118.2	O8—C23—C24	122.67 (9)
C2—C1—H1	118.2	O9—C23—C24	112.57 (9)
N3—C16—C15	122.25 (10)	C24^i^—C24—C23	122.28 (12)
N3—C16—H16	118.9	C24^i^—C24—H24	118.9
C15—C16—H16	118.9	C23—C24—H24	118.9
C16—C15—C14	119.04 (9)	O6—C22—O7	124.52 (9)
C16—C15—H15	120.5	O6—C22—C21	120.91 (9)
C14—C15—H15	120.5	O7—C22—C21	114.57 (9)
C15—C14—C18	118.86 (9)	C20—C21—C22	123.99 (9)
C15—C14—C13	120.32 (9)	C20—C21—H21	118.0
C18—C14—C13	120.81 (9)	C22—C21—H21	118.0
C17—C18—C14	118.51 (10)	C21—C20—C19	123.71 (9)
C17—C18—H18	120.7	C21—C20—H20	118.1
C14—C18—H18	120.7	C19—C20—H20	118.1
N3—C17—C18	122.46 (9)	O4—C19—O5	124.32 (9)
N3—C17—H17	118.8	O4—C19—C20	121.40 (9)
C18—C17—H17	118.8	O5—C19—C20	114.27 (9)

Symmetry code: (i) −*x*+1, −*y*+2, −*z*.

### Hydrogen-bond geometry (Å, º)

**Table:**

*D*—H···*A*	*D*—H	H···*A*	*D*···*A*	*D*—H···*A*
O9—H9*A*···N3	1.030 (19)	1.576 (19)	2.6047 (12)	176.1 (17)
O5—H5···N1	1.03 (2)	1.57 (2)	2.5952 (12)	172.0 (18)
O7—H7···N2	1.050 (19)	1.54 (2)	2.5826 (12)	172.9 (18)
